# Supplementary material for: Pavlovian-To-Instrumental Transfer and Alcohol Consumption in Young Male Social Drinkers: Behavioral, Neural and Polygenic Correlates
Source: J Clin Med. 2019 Aug 8;8(8):1188. doi: 10.3390/jcm8081188 (PMC6723486; doi:10.3390/jcm8081188)
Supplement: Supplementary file 1 [file jcm-08-01188-s001.pdf]

Supplement

Representativeness of our study cohort

Participants were descriptively comparable to similar cohorts drawn from the German general population regarding monthly per-capita income, DSM-IV nicotine dependence, alcohol consumption (except for an underrepresentation of alcohol-abstinent persons and an overrepresentation of persons drinking only small amounts of alcohol due to our inclusion criteria), DSM-IV alcohol abuse and BMI (see Table S1).

Table S1. Comparing our study cohort to a reference population.

Variable	Study cohort (males, 18 years, *n*=191)	Reference population	
Monthly per-capita income	*M*=861 EUR ± 375 EUR (*n*=116)	*M*=906 to 1130 EUR	German households with children under age 18[1]
DSM-IV Nicotine dependence (past year)	7%	10%	males, 18-20 years[2]
Alcohol consumption (past year)			
Abstinence	0% (*Exclusion criterion)*	15%	males, 18-20 years[2]
0.5-24 g/day	86%	61%	
>25-60 g/day	12%	14%	
>60-120 g/day	<1%	2%	
>120 g/day	<1%	1%	
Mean g/week	85g	92 g	males, 18-24 years[2]
DSM-IV alcohol abuse (lifetime)	6%	8%	males, 18-20 years[2]
BMI			males, 18-29 years[3]
M ± *SD*	22.3 ± 3.1	24.5 ± -	
underweight (<18.5)	6%	3%	
normal (18.5-<25)	76%	62%	
overweight (25-<30)	14%	27%	
Adipositas (≥30)	3%	9%	

Note. Data are for descriptive purposes only. Reference populations come from different studies.

Measures of alcohol consumption

To characterize participants' drinking behavior and how this relates to polygenic risk, we calculated a sum score of drinking variables using information acquired with the CIDI interview[4, 5]: age of 1st drink, age of 1st time being drunk, estimated average alcohol consumption in past year (g alc/day), average alcohol consumption per drinking occasion in the past year (g alc), age of 1st binge-drinking event, number of binge-drinking events lifetime, and average alcohol consumption per binge-drinking event in the past year (g alc). Binge-drinking was defined as the consumption of at least five drinks (≥60g alc) on one occasion. To increase reliability of the indicator of alcohol drinking behavior, we did not use the single CIDI items but calculated a sum score (*drink score*) from the z-scaled CIDI items with higher values indicating greater alcohol consumption[6]. Unlike the categorical high- versus low-risk drinker classification by the WHO, the resulting score is a continuous measure of alcohol intake that can be used for linear correlations with the polygenic risk score for alcohol consumption.

Moreover, the WHO suggests a second criterion for chronic harm of alcohol consumption based on the average alcohol intake per day[7] as assessed with the CIDI interview[4, 5]. However, 95.8 % of our sample fell into the low-risk group according to this criterion rendering our data unsuitable to examine associations of chronic risk of alcohol intake with PIT. Given this distribution of chronic risk and the young age and relatively brief period of alcohol consumption (on average two years) of our participants, chronic risk was of secondary interest to us and, therefore, we focused on comparisons between participants with low- versus high-risk drinkers for acute alcohol-related problems[7] in our analyses as described in the main text.

Preprocessing of fMRI data

FMRI data were pre-processed using Nipype[8]. Correction for differences in slice time acquisition was performed. Voxel-displacement maps were estimated based on acquired field maps. All images were realigned to correct for motion, distortion and their interaction. After coregistration of the individual structural image to the individual mean EPI, the structural image was spatially normalized and normalization parameters were applied to all EPIs. Finally, images were spatially smoothed with a Gaussian kernel of 8 mm full width at half maximum. During individual statistical analyses, data were high-pass filtered with a cut-off of 128 s. An event-related analysis was applied on two levels using the general linear model approach as implemented in SPM12.

Sample characteristics

In Table S2 the sample characteristics for the two groups of low- (n = 97) and high-risk drinkers (n = 94) are displayed.

Table S2. Sample characteristics for the two groups of low- and high-risk drinkers.

	**Low-risk drinkers (97)**		**High-risk drinkers (94)**		***p***
	**mean**	**SD**	**mean**	**SD**	
Age	18.4	0.1	18.4	0.2	.96[a]
SES	-0.03	1.4	0.03	1.9	.83[a]
Verbal intelligence (MWT-B)	98.1	4.9	97.5	5.5	.26[b]
Pure alcohol in kg consumed during lifetime	4.2	4.0	12.7	11.2	<.001[b]
DSM-IV alcohol abuse (n)	1	-	12	-	-
Alcohol craving (OCDS)	3.2	2.8	4.2	3.3	.009[b]
Alcohol use problems (ADS)	3.9	4.1	5.9	4.1	<.001[b]
Nicotine dependence severity (FTND)	0.2	1.1	0.2	0.7	.71[a]

Note. SES – socio-economic status: computed as the sum of z-transformed social status, household income and inverse personal debt scores[9]; MWT-B - Mehrfachwahl-Wortschatz-Intelligenztest, test for verbal intelligence[10]; OCDS - Obsessive Compulsive Drinking Scale, alcohol craving[11]; ADS - Alcohol Dependence Severity[12]; FTND - Fagerström Test for Nicotine Dependence, nicotine dependence severity[13]. [a] two-sample *t*-test, two-tailed; [b] Wilcoxon rank sum test, two-tailed.

Behavioral PIT effect by group (high- versus low-risk drinkers according to WHO)

We observed a stronger PIT effect in high-risk drinkers as indicated by a significant interaction effect of group (low- versus high-risk drinkers) and Pavlovian background value (ranging from -2€ to +2€) on number of button presses. Moreover, both groups learned the instrumental contingencies equally well as we found a significant main effect of instrumental condition (collect versus not-collect), but no interaction between instrumental condition and group. See Table S3 for all main and interaction effects of the regression model.

Table S2. Regression coefficients indicating the influence of Pavlovian background conditions (-2€, -1€, 0€, +1€, +2€), instrumental conditions (collect versus not-collect) and drinking group

**Coefficient**	**Estimate(SE)**	***z***	***p***
Intercept	0.85(0.07)	11.53	<.001
Pavlovian background value	0.12(0.02)	7.27	<.001
Instrumental task (collect versus not collect)	2.27(0.17)	13.41	<.001
Group (low versus high risk drinkers)	-0.04(0.14)	-0.30	.76
Pavlovian background value * group	0.09(0.03)	2.70	<.009
Instrumental task * group	-0.20(0.34)	-0.61	.54

Moreover, we controlled our results for smoking severity as measured by the FTND[13] and still observed a similar pattern (see Table S4).

Table S4. Regression coefficients indicating the influence of Pavlovian background conditions (-2€, -1€, 0€, +1€, +2€), instrumental conditions (collect versus not-collect) and drinking group controlled for smoking severity.

**Coefficient**	**Estimate(SE)**	***z***	***p***
Intercept	0.85(0.07)	11.54	<.001
Pavlovian background value	0.12(0.02)	7.32	<.001
Instrumental task (collect versus not collect)	2.27(0.17)	13.41	<.001
Group (low versus high risk drinkers)	-0.04(0.14)	-0.30	.76
Smoking severity	-0.02(0.08)	-0.22	.82
Pavlovian background value * group	0.09(0.03)	2.73	<.01
Instrumental task * group	-0.20(0.34)	-0.61	.54
Pavlovian background value * smoking severity	-0.02(0.02)	-1.02	.31
Instrumental task * smoking severity	-0.05(0.18)	0.29	.77
group * smoking severity	-0.05(0.15)	-0.33	.74
Pavlovian background value * group * smoking severity	-0.03(0.04)	-0.94	.35
Instrumental task *group * smoking severity	0.23(0.37)	0.62	.53

Behavioral PIT effect in whole group

With respect to the overall learning effects in the whole group of 191 male 18-year old subjects, our regression analysis revealed a main effect of collect/not collect ($\beta = 2.27$, $SE = 0.17$, $p < .001$): all participants showed a higher number of button presses during approach compared to non-approach trials, indicating successful instrumental learning. Moreover, we observed a main effect of Pavlovian background on the number of button presses ($\beta = 0.12$, $SE = 0.02$, $p < .001$), indicating a significant influence of background cues on instrumental response for the whole group (see Figure S1).

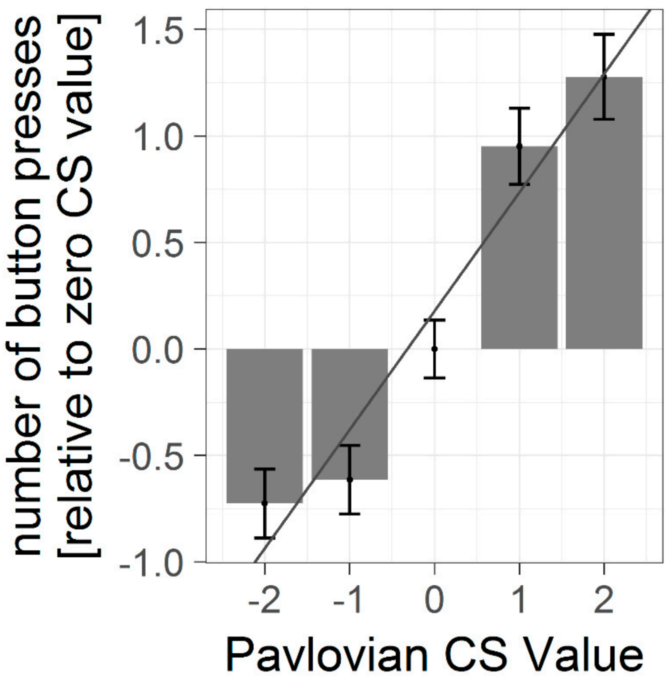

Figure S1. PIT effect in the whole sample (n=191). Number of button presses are displayed relative to the zero (0€) condition. Grey line reflects the group regression slope.

Neural CS effect

To explore the pure effect of CS (compound of fractal-like visual stimulus and tone in the background) during our PIT paradigm, we assessed on the second level the CS value across the whole group and again performed a small volume analysis using a ROI encompassing both the NAcc and the amygdala. We found no significant activation within the left (t = 0.79, p_{SVC} = .82, x = -22, y = -8, z = -22) nor right amygdala (t = 0.9, p_{SVC} = .79, x = 24, y = -8, z = -10). However, we found a CS value related activation in the right NAcc (t = 3.25, p_{SVC} = .041, k = 1, x = 12, y = 10, z = -8, see Figure S2). This activation was elicited by passively viewing CSs without explicit task relevance for the subjects as instructed by us; thus, the effect is not directly comparable with alcohol-related cue-reactivity or monetary incentive delay tasks, where stimuli predict possible win or loss in accordance with fast responses.

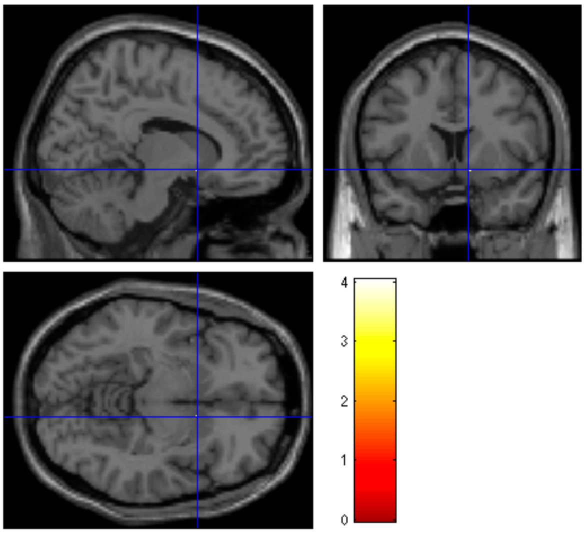

Figure S2. Pure CS effect during the PIT task. This effect was significant for the whole imaging group of n=139 subjects in the right NAcc only (p_{SVC} = .041).

Exploratory whole brain analysis of PIT effect

The following Table S5 aims to give an overview of the exploratory whole brain analysis of the PIT-effect. We used a threshold of $p_{uncorr} < .001$ and cluster size $k = 10$.

Table S5. Exploratory whole brain analysis of PIT-related activations.

**set**		**cluster**				**peak**					**coordinates in mm**			**Automated anatomical labeling (AAL)**
p	*c*	$p_{(FWE\text{-}corr)}$	$p_{(FDR\text{-}corr)}$	*k*	$p_{(unc)}$	$p_{(FWE\text{-}corr)}$	$p_{(FDR\text{-}corr)}$	*T*	*Z*	$p_{(unc)}$	x	y	z	
.49	3	.82	.92	31	.44	.52	.90	3.78	3.68	< .001	24	-6	-6	R Pallidum
		.74	.92	44	.35	.62	.90	3.69	3.60	< .001	54	-8	-6	R Superior Temporal Gyrus
		.88	.92	20	.54	.75	.90	3.56	3.47	< .001	-32	-40	10	L Hippocampus

Multi-level statistical approach including multi-modal data

We conducted an additional generalized linear mixed-effects analysis including multi-modal data as additional predictors (behavioral PIT, alcohol drinking risk groups and genetic information) using the subjects that have full data sets (n = 115, see Table S6). This analysis confirmed our results of enhanced PIT effects in high- compared to low-risk drinking subjects (trend-wise due to lower statistical power in the smaller cohort, interaction effect between Pavlovian background value and group, $p = .053$). Moreover, we observed enhanced PIT effects with stronger amygdala activation (interaction effect between Pavlovian background value and amygdala activation, $p < .001$) and higher polygenic risk for alcohol consumption (interaction effect between Pavlovian background value and PRS, $p = .019$). All tests were conducted two-tailed.

Table S6. Generalized linear mixed-effects model for predicting number of button presses as a function of instrumental task (collect versus not collect) and Pavlovian background stimulus value (-2€, -1€, 0€, +1€, +2€). Moreover, this analysis includes additional predictors, namely alcohol risk group, neural PIT effect and polygenic risk (n = 115).

**Coefficient**	**Estimate (SE)**	***z***	***p***
intercept	1.22(0.31)	3.91	< .001
Pavlovian value of background	-0.01(0.05)	-0.19	.846
Instrumental task (collect versus not collect)	1.73 (0.71)	2.42	.016
WHO risk group (low versus high risk alcohol drinkers)	-0.29(0.20)	-1.49	.138
Amygdala activation	0.03(0.11)	0.31	.760
PRS (z)	0.17(0.10)	1.76	.079
Pavlovian value * instrumental task	-0.03(0.03)	-0.93	.354
Pavlovian value * WHO risk group	0.06(0.03)	1.94	.053
Pavlovian value * amygdala activation	0.07(0.02)	4.09	< .001
Pavlovian value * PRS (z)	0.04(0.02)	2.35	.019
Instrumental task * WHO risk group	0.52(0.45)	1.15	0.250
Instrumental task * amygdala activation	-0.23(0.25)	-0.92	0.360
Instrumental task * PRS	-0.41(0.22)	-1.83	.068
Pavlovian value * instrumental task * WHO risk group	-0.03(0.02)	-1.50	0.133
Pavlovian value * instrumental task * amygdala activation	-0.03(0.01)	-3.18	0.002
Pavlovian value * instrumental task * PRS (z)	-0.03(0.01)	-2.82	0.005

References

1. Statistisches Bundesamt. Einkommen und Einnahmen sowie Ausgaben privater Haushalte, (Laufende Wirtschaftsrechnungen): Fr.Bundesgebiet/N.Länder,Jahre (bis 2012), Haushaltsnettoeinkommensklassen https://www-genesis.destatis.de/genesis/online, 2012.

2. Kraus L, Pabst A, Gomes de Matos E, Piontek D. Kurzbericht Epidemiologischer Suchtsurvey 2012. Tabellenband: Prävalenz des Tabakkonsums und der Nikotinabhängigkeit nach Geschlecht und Alter im Jahr 2012. IFT Institut für Therapieforschung: München, 2013.

3. Mensink GB, Lampert T, Bergmann E. Overweight and obesity in Germany 1984-2003. *Bundesgesundheitsblatt, Gesundheitsforschung, Gesundheitsschutz* 2015; **48:** 1348-1356.

4. Jacobi F, Mack S, Gerschler A, Scholl L, Hofler M, Siegert J *et al.* The design and methods of the mental health module in the German Health Interview and Examination Survey for Adults (DEGS1-MH). *International journal of methods in psychiatric research* 2013; **22**(2)**:** 83-99.

5. Wittchen H-U, Pfister H. *DIA-X-Interviews: Manual Für Screening-Verfahren Und Interview; Interviewheft Längsschnittuntersuchung (DIA-X-Lifetime); Ergänzungsheft (DIA-X-Lifetime); Interviewheft Querschnittuntersuchung (DIA-X-12 Monate); Ergänzungsheft (DIA-X-12 Monate); PC-Programm Zur Durchführung Des Interviews (Längs- Und Querschnittuntersuchung); Auswertungsprogramm*. Swets & Zeitlinger: Frankfurt, 1997.

6. Nebe S, Kroemer NB, Schad DJ, Bernhardt N, Sebold M, Muller DK *et al.* No association of goal-directed and habitual control with alcohol consumption in young adults. *Addiction biology* 2017.

7. World Health Organization DoMhaSD. *International guide for monitoring alcohol consumption and related harm. Technical Document WHO/MSD/MSB/00.4*. World Health Organisation: Geneva, 2000.

8. Gorgolewski K, Burns CD, Madison C, Clark D, Halchenko YO, Waskom ML *et al.* Nipype: a flexible, lightweight and extensible neuroimaging data processing framework in python. *Frontiers in neuroinformatics* 2011; **5:** 13.

9. Schmidt LG, Gastpar M, Falkai P, W G. *Evidenzbasierte Suchtmedizin*. Behandlungsleitlinie Substanzbezogene Störungen: Köln, 2006.

10. Lehrl S, Triebig G, Fischer B. Multiple choice vocabulary test MWT as a valid and short test to estimate premorbid intelligence. *Acta neurologica Scandinavica* 1995; **91**(5)**:** 335-345.

11. Mann K, Ackermann K. Die OCDS-G: Psychometrische Kennwerte der deutschen Version der Obsessive Compulsive Drinking Scale. *Sucht* 2000; **46:** 90-100.

12. Skinner HA, Horn JL. *Alcohol Dependence Scale (ADS): Users Guide*. Addiction Research Foundation: Toronto, 1984.

13. Heatherton TF, Kozlowski LT, Frecker RC, Fagerstrom KO. The Fagerstrom Test for Nicotine Dependence: a revision of the Fagerstrom Tolerance Questionnaire. *British journal of addiction* 1991; **86**(9)**:** 1119-1127.
